# First Identification of Reinfection by a Genetically Different Variant of SARS-CoV-2 in a Homeless Person from the Metropolitan Area of Santiago, Chile

**DOI:** 10.1155/2022/3859071

**Published:** 2022-04-27

**Authors:** Claudio Acuña-Castillo, Mabel Vidal, Ailen Inostroza-Molina, Eva Vallejos-Vidal, Roberto Luraschi, Maximiliano Figueroa, Carlos Barrera-Avalos, Rodrigo Vera, Sergio Vargas, Daniel Valdes, Kevin Maisey, Mónica Imarai, Elías Leiva-Salcedo, Alejandro Escobar, Sebastián Reyes-Cerpa, Alexis Gaete, Ricardo Palma-Vejares, Dante Travisany, Leonel E. Rojo, Felipe E. Reyes-López, Ana María Sandino

**Affiliations:** ^1^Centro de Biotecnología Acuícola, Facultad de Química y Biología, Universidad de Santiago de Chile, Santiago, Chile; ^2^Departamento de Biología, Facultad de Química y Biología, Universidad de Santiago de Chile, Santiago, Chile; ^3^Department of Computer Science, University of Concepcion, Concepción 4070409, Chile; ^4^Centro de Nanociencia y Nanotecnología CEDENNA, Universidad de Santiago de Chile, Santiago, Chile; ^5^Facultad de Medicina Veterinaria y Agronomía, Universidad de Las Américas, Santiago, Chile; ^6^Hospital de Urgencia Asistencia Pública (HUAP), Santiago, Sección Biología Molecular, Laboratorio Clínico HUAP, Chile; ^7^Laboratorio Biología Celular y Molecular, Instituto de Investigación en Ciencias Odontológicas, Facultad de Odontología, Universidad de Chile, Santiago, Chile; ^8^Centro de Genómica y Bioinformática, Facultad de Ciencias, Universidad Mayor, Santiago, Chile; ^9^Escuela de Biotecnología, Facultad de Ciencias, Universidad Mayor, Santiago, Chile; ^10^Laboratorio de Bioinformática y Expresión Génica, Instituto de Nutrición y Tecnología de los Alimentos, Universidad de Chile, Santiago, Chile; ^11^Fondap Center for Genome Regulation, Facultad de Ciencias, Universidad de Chile, Santiago, Chile; ^12^Centro de Modelamiento Matemático UMI-CNRS 2807, Facultad de Ciencias Físicas y Matemáticas, Universidad de Chile, Santiago, Chile; ^13^Inria Chile Research Center, Santiago, Chile; ^14^Department of Cell Biology, Physiology, and Immunology, Universitat Autònoma de Barcelona, Bellaterra, Spain

## Abstract

The identification and tracking of SARS-CoV-2 infected patients in the general population are essential components of the global strategy to limit the COVID-19 viral spread, specifically for maintaining traceability and suppressing the resurgence of local outbreaks. Public health programs that include continuous RT-qPCR testing for COVID-19 in the general population, viral sequencing, and genomic surveillance for highly contagious forms of the virus have allowed for the identification of SARS-CoV-2 infections and reinfections. This work identified SARS-CoV-2 reinfection in a homeless person, which occurred 58 days after the first COVID-19 diagnosis. Genomic sequencing identified a different Nextstrain classification clade (20A and 20B) and PANGO lineage, with a divergence of 4 single nucleotide variants (SNVs) in S and ORF1ab genes, suggesting reinfection by different viral variants. This study is the first from the great metropolitan area of Santiago, Chile, one of the top ten countries in the world to live during the COVID-19 pandemic. We support the importance of performing intensive genomic surveillance programs in the whole population and high-risk groups, such as homeless people, nearly 20 thousand people in Chile, and have limited access to health care services and poor viral traceability.

## 1. Introduction

The COVID-19 pandemic caused by the severe acute respiratory syndrome coronavirus 2 (SARS-CoV-2) has caused more than 260 M infections and 5.2 M deaths worldwide [1]. The topmost practical approaches to control this pandemic include vaccination, social distance, and common barriers (i.e., face masks) [2]. The active search of COVID-19 by RT-qPCR on a large scale and the traceability of positive patients are valuable tools towards limiting new outbreaks of the virus with highly contagious variants that can generate reinfections [3, 4]. In a study by Hall et al. (2021), healthcare workers under continuous testing in England showed that reinfections were unusual, at around 6–7 per 100,000 patients. However, they carry high viral loads in their throats, increasing the risk of transmitting the virus [5]. SARS-CoV-2 can cause COVID-19 reinfections with different combinations of mutations among different clades, as demonstrated in a recent study with Indian healthcare workers, where nine mutations were found in reinfected individuals compared to the ancestral SARS-CoV-2 [6]. Reinfection with different viral lineages can occur even in patients with a complete vaccine scheme, displaying severe acute symptoms [7]. This possibility of reinfection in vaccinated patients jeopardizes the effective control of the COVID-19 pandemic, as vaccine programs are the cornerstone of all governmental strategies to control COVID-19 spread. Therefore, reinfection events are essential to consider and analyze. The present research shows the genomic evidence of reinfection with a different viral lineage in a homeless female, detected in the greater metropolitan area of Santiago, Chile. Despite being a retrospective study, this supports the importance of maintaining a continuous analysis of the population, emphasizing those who live with limited access to medical services and little traceability to health authorities, such as the homeless population, which numbers around 20 thousand people in Chile.

## 2. Methods

### 2.1. Sample Description

A 54-year-old homeless female with clinical records of alcoholism and drug addiction but no other chronic diseases was referred from the mental health service for SARS-CoV-2 RT-qPCR testing in a community health care center. The patient tested positive for SARS-CoV-2 on June 6^th^. However, she was not quarantined and was untraceable until June 15^th^, when she was admitted to a local overnight shelter managed by public social services. The patient registered mild symptoms during these ten days. On August 3^rd^, the patient requested medical attention for an infected foot wound in a public healthcare center. The patient tested positive for SARS-CoV-2 again by RT-qPCR and positive for IgG using a rapid antigen test. The patient showed no COVID-19 related symptoms and was medically discharged on August 14th.

### 2.2. Sample and RT-qPCR Assay

Nasopharyngeal swab samples (NPSs) were obtained from the patient by routine protocols implemented by Health COVID-19 government policy. Samples were transported to the Universidad de Santiago Laboratory in a viral transport medium (GenoSur, Chile). Total RNA was extracted as previously described [8]. The detection of SARS-CoV-2 was carried out using the ORF1ab gene probe (TaqMan™ 2019nCoV Assay Kit v1 (Thermo Fisher Scientific, Reference code: A47532) using a one-step strategy as previously reported by our group [8].

### 2.3. Sequencing and Analysis

cDNA synthesis was performed from two-positive samples using the High-Capacity RNA-to-cDNA kit (Applied Biosystems) and amplified by the ARTIC v3 protocol [9] using the 98 primers set with the GoTaq Green Master Mix enzyme (Promega). The genomic library was constructed using the Ligation Sequencing Kit SQK-LSK109 (Oxford Nanopore) and processed and sequenced independently (without barcodes) to increase and know the exact number of readings per sample. Sequencing reached 1 Gigabase (Gb) (approximately 3 hours of sequencing) without the base-calling process. The nanopore sequencing was transformed to fastq format using the ONT Guppy-based caller Guppy (https://nanoporetech.com) (CPU mode). All reads were quality checked and assembled using ARTICMINION (https://artic.network/ncov-2019). The consensus assembly genomes were aligned to the reference SARS-CoV-2 genome using Nucmer (MUMmer), and SNPs were predicted using the Nucmer tool to show SNPs [10]. Then, SNPs were annotated using SnpEff (SNPEFF) within the SARS-CoV-2 database [11]. All computations were run at the National Laboratory for High-Performance Computing (https://www.nlhpc.cl) [12]. We performed a second bioinformatic analysis using an independent process and open-source tools. Sequence pairs were aligned to the SARS-CoV-2 reference genome using Bowtie 2 version 2.3. Variants were called Freebayes version 1.3.5 with a ploidy setting of 1 and a minimum allele frequency of 0.70. The coverage statistics were performed using VCFtools version 0.1.17 to determine the mutation rate between the samples. In addition, the Nextstrain clade assignment was performed using NextClade version 1.9.0 to compare and visualize our samples globally. The PANGO lineage was analyzed with the pangolin software [13]. The Clustal12.1 tool was used for identity analysis between the two samples. The BioProyect database PRJNA768603: 0606–201(SAMN22047842) and 0308–063 (SAMN22047843) from BioSample data are available in the NCBI. The original virus sequence used to compare both samples was as follows: severe acute respiratory syndrome coronavirus 2 isolate Wuhan-Hu-1, ACCESSION MN908947, March 2020, from the GeneBank database.

## 3. Results

**Figure 1:**
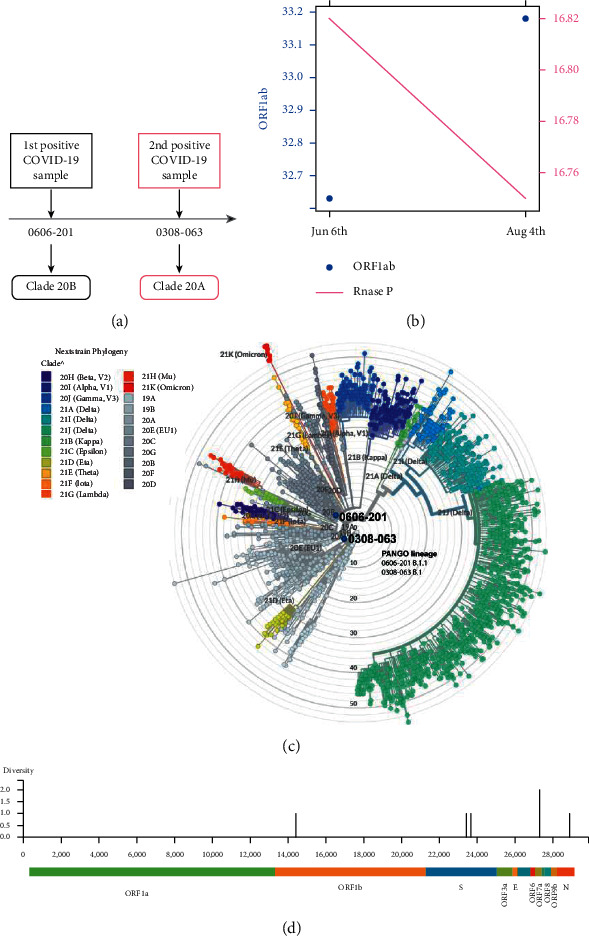
Genomic analysis of reinfected homeless patients. (a) Timeline shows the first (June 6th) and second (Aug 3rd) positive diagnosis by RT-qPCR for SARS-CoV-2. Nextstrain viral classification clade is shown for both samples. (b) RT-qPCR data using ORF1ab and RNase P (internal cellular control) probes for the homeless patient from nasopharyngeal swab sampling was obtained on June 6^th^ (0606-201) and 58 days later, on August 3^rd^ (0308-063). Cq-values are shown for ORFab (blue point) on the left and RNase P (pink line) on the right. (c) Clade structure of SARS-CoV-2 defined by Nexclade version 1.9.0 in Nextstrain classification: sample 0606-201 in clade 20B and 0308-063 in clade 20A. PANGO lineage classification by pangolin tool (0606-201; B.1 and 0308-063; B.1.1). (d) Genetic diversity plot of both pieces referred to the ancestral SARS-CoV genome. The highest diversity is present at ORF1b, ORF7a, S, and N.

This patient was a mild-symptomatic female subject who had positive NPSs for SARS-CoV-2 on June 6^th^ (0606-201) and 58 days later, on August 3^rd^ (0308-063), without COVID-19 symptom (Figure 1(a)). The ORF1ab amplification curve showed Cq = 32.63 and Cq = 33.18, respectively (Figure 1(b)). In both samples, the RNase P amplification was quite similar (Cq = 16.82; Cq = 16.75, respectively) (Figure 1(b)). The detection limit for ORF1ab, as considered by our laboratory and described previously [14], confirmed the positive diagnosis for COVID-19 for both samples. The phylogenetic analysis showed that 0606-201 and 0308-063 samples were members of Nextstrain clades 20B and 20A, respectively (Figure 1(c)). Between March and June 2020, viral samples belonging to clades 19A and 20 were reported, according to the Chilean GISAID database. From a global perspective, the most predominant clades between March and June 2020 were 19A, 19B, 20A, 20B, 20D, 20C, and 20F. For sample 0308-063, we observed that only clade 20B was reported in Chile. While between June and September 2020, the most represented clades were 19A, 19B, 20A, 20B, 20D, 20F, 20E (EU1), 20C, and 20H (Beta, V2) globally (Supplementary Figure 1) [15]. We observed a divergence of 6 and 4 single nucleotide variants (SNVs) for the 0606-201 and 0308-063 samples, respectively, when they were compared with the ancestral SARS-CoV-2 genome (acc. MN908947) (Figure 1(d), Table 1). The sample 0606-201 showed six SNVs (23403 A ⟶ G, 23664C ⟶ T, 27290A ⟶ G, 28881G ⟶ A, 28882G ⟶ A, and 28883G ⟶ C), sharing 2 SNVs with the 0308-063 sample. The remaining 4 SNVs were not observed in the 0308-063 sample, and two new SNVs were identified (S:23409 A ⟶ G and ORF8: 28217T ⟶ C) (Table 1). SNVs of the specimen 0606-201 were in the genes S (spike), N (nucleocapside), and ORF (open reading frame) 6. The sample 0308-063 shared two SNVs with sample 0606-201, located in the S and ORF6 genes, while no SNVs were detected in the N gene, and a new mutation in the ORF8 gene was identified in 0308-063 (Table 1; Figure 1(d)), suggesting reinfection by another virus linage.

## 4. Discussion

The active search for COVID-19 patients on a large scale allows the timely isolation of infected patients and the identification of unusual SARS-CoV-2 reinfections. The study identified reinfection with SARS-CoV-2 performed in one of the largest cities of South America, Santiago, Chile. A negative RT-qPCR between both positive samples is necessary to confirm reinfection, as previously reported [16, 17], since in some scenarios, a coinfection is possible when the first infection is still developing [18]. We rule out the mutation and persistence of the same virus since at least six mutations differentiate the virus from the first to the second sample in less than two months, where the virus's mutation rate is two SNVs per month [19]. These mutations are related to different Nextstrain and PANGO lineages.

Our results support the importance of active search and genomic surveillance for COVID-19 on a large scale. It allows for the timely isolation of infected patients and the identification of patients presenting viral reinfection and new variants. Viral reinfections may be associated with weakened humoral immunity in the first months after a natural SARS-CoV-2 infection [20, 21]. Our study suggests that the homeless population may be at high risk because a decreased immunity status has been found as an underlying condition in this group of people, such as malnutrition and drug abuse [22, 23]. Such conditions make them more vulnerable to infections and susceptible to more severe manifestations of the disease than healthy individuals [24]. More importantly, according to other studies, due to the lack of traceability in homeless people, these reinfections by other SARS-CoV-2 lineages could generate new outbreaks of infections with the circulation of new variants even in the vaccinated population [7]. Although we present only a clinical antecedent, this data provide the basis to alert and suggest continuous traceability to the homeless population to control and analyze SARS-CoV-2 and its variants to avoid viral spread, as it was already done in Brussels, Belgium [25]. In addition, their shelters are generally crowded and can generate outbreaks of infection and the development of possible new variants, as another study suggests [26]. Finally, although it is necessary to analyze more antecedents to draw more remarkable conclusions, our report emphasizes the importance of establishing preventive public health policies in the whole population to control SARS-CoV-2 and future pandemics.

## Figures and Tables

**Table 1:** The single nucleotide variants and amino acid substitutions of both samples (0606-201 and 0308-063) of the complete genome of SARS-CoV-2 Wuhan-1.

Genome location	0606-201	0308-063	SARS-CoV-2 gene location	Amino acid substitutions
23403	A->G	A->G	S	D614G
23664	C->T	None	S	A701V
23409	None	A->G	S	N616S
27290	A->G	A->G	ORF6	D30G
28217	n/a	T->C	ORF8	None
28881	G->A	None	N	R203K
28882	G->A	None	N	G204G
28883	G->C	None	N	None

## Data Availability

The data that support the findings of this study are available in BioProyect metadata from the NCBI database (http://www.ncbi.nlm.nih.gov/bioproyect) under accession number PRJNA768603. The BioSample data are SAMN22047842 (for sample 0606-201) and SAMN22047843 (for sample 0308-063).

## Supplementary Materials

Supplementary Materials Samples were reported in the GISAID database. 0606-20 sample 1: until June in Chile (March to June 2020, on the left), clade 19B and 20B were reported. In global (on the right), the clade 19A, 19B, 20A, 20B, 20D, 20F, and 20C were reported. For 0308-063 sample: between July and September 2020 in Chile (on the left) only clade 20B was reported. While in global (on the right), the clades 19A, 19B, 20A, 20B, 20D, 20F, 20E (EU1), 20C, and 20H (Beta, V2) were reported.
