# Supplementary material for: First Identification of Reinfection by a Genetically Different Variant of SARS-CoV-2 in a Homeless Person from the Metropolitan Area of Santiago, Chile
Source: J Environ Public Health. 2022 Apr 27;2022:3859071. doi: 10.1155/2022/3859071 (PMC9068328; doi:10.1155/2022/3859071)
Supplement: Supplementary Materials — Samples were reported in the GISAID database. 0606-20 sample 1: until June in Chile (March to June 2020, on the left), clade 19B and 20B were reported. In global (on the right), the clade 19A, 19B, 20A, 20B, 20D, 20F, and 20C were reported. For 0308-063 sample: between July and September 2020 in Chile (on the left) only clade 20B was reported. While in global (on the right), the clades 19A, 19B, 20A, 20B, 20D, 20F, 20E (EU1), 20C, and 20H (Beta, V2) were reported. [file 3859071.f1.docx]

**Supplementary Figure**

**
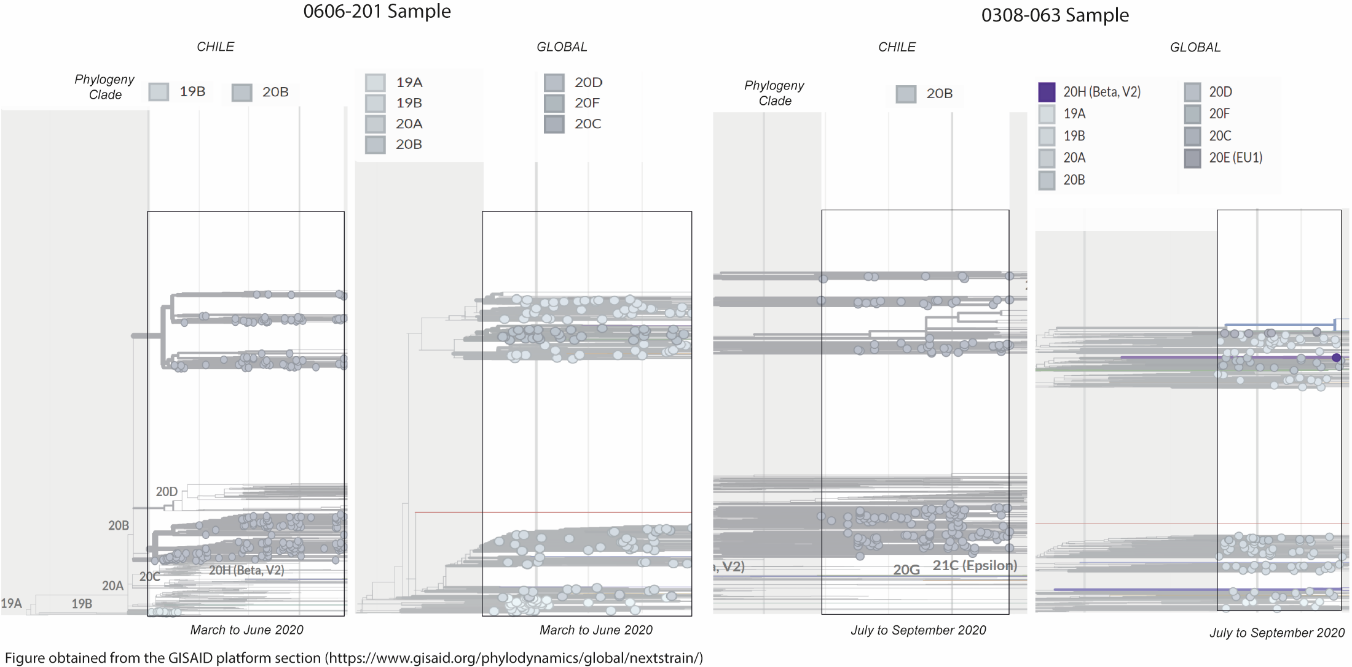
**

**Supplementary Figure 1.** Samples were reported in the GISAID database. 0606-20 sample 1: until June in Chile (March to June 2020, on the left), clade 19B and 20B were reported. In global (on the right), the clade 19A, 19B, 20A, 20B, 20D, 20F and 20C were reported. For 0308-063 sample: between July and September 2020 in Chile (on the left) only clade 20B was reported. While in global (on the right), the clades 19A, 19B, 20A, 20B, 20D, 20F, 20E (EU1), 20C and 20H (Beta, V2) were reported.
